# Type I Lip Patterns among Medical Students of a Medical College

**DOI:** 10.31729/jnma.8286

**Published:** 2023-10-31

**Authors:** Kabir Shrestha, Samarika Dahal, Radha Baral, Abishikha Neupane

**Affiliations:** 1 Maharajgunj Medical Campus, Maharajgunj, Kathmandu, Nepal; 2 Department of Oral Pathology and Forensic Dentistry, Maharajgunj Medical Campus, Maharajgunj, Kathmandu, Nepal

**Keywords:** *biometric identification*, *forensic science*, *lips*, *medical students*, *photography*

## Abstract

**Introduction:**

Identity and identification have long been a source of interest and concern in forensic dentistry, whether in the context of a criminal investigation or the identification of a deceased person. Lip print has demonstrated a high level of potential as one of the best options, as well as its usage as supporting evidence. The aim of this study was to find out the prevalence of Type I lip patterns among medical students of a medical college.

**Methods:**

This descriptive cross-sectional study was done among medical students of a medical college from 30 December 2021 to 30 February 2022 after obtaining ethical approval from the Institutional Review Committee. The lipstick was applied in a thin layer uniformly, and the impression was taken with the help of the cellophane tape. The specimens were analyzed and classified based on Tsuzuki and Tsuchihashi's classification. A convenience sampling method was used. The point estimate was calculated at a 95% Confidence Interval.

**Results:**

Among 120 medical students, the prevalence of type I lip pattern was 48 (40%) (31.23-48.77, 95% Confidence Interval). Among them, 26 (54.17%) were males and 22 (45.83%) were females.

**Conclusions:**

The prevalence of Type I lip pattern among medical students was higher than in other studies done in similar settings.

## INTRODUCTION

Lip prints are a pattern of lines that develop on the red part of the lips (vermilion) or the transitioning area of the lip. Each person's pattern of small wrinkles in the vermilion of the lips is distinct and identical.^1,2^ There are numerous variations in the lip prints, including variances in the number of lines, thickness, length, branching, location, and combinations.^3^ A cohort study showed that the lip pattern does not change over time, and remains the same.^4^

Lip prints resemble fingerprints in appearance and can be utilized in the identification of an individual and add value in forensic dentistry in the context of criminal investigation or identification of a deceased person.^5^

The aim of this study was to find out the prevalence of Type I lip patterns among medical students of a medical college.

## METHODS

A descriptive cross-sectional study was conducted in the Department of Oral Pathology and Forensic Dentistry of Maharajgunj Medical Campus, Maharajgunj, Kathmandu, Nepal from 30 December 2021 to 30 February 2022. The ethical approval was taken from the Institutional Review Committee of the same institute [Reference number: 230(6-11) E2 078/079]. Medical students aged between 18 to 25 years with normal transition zones between mucosa and skin, and without any pathological conditions like swelling, ulcers or were included in the study. Those students who had not given consent and had allergic conditions to lipstick were excluded from the study. The convenience sampling method was used. The sample size was calculated using the following formula:


$$\begin{array}{ll} \text{n} & ={\text{Z}}^{2}\times \frac{\text{p}\times \text{q}}{{\text{e}}^{\text{2}}}\\ & =1.96^{2}\times \frac{0.34\times 0.66}{0.06^{2}}\\ & =267 \end{array}$$

Where,

n = minimum required sample size
Z = 1.96 at 95% Confidence Interval (CI)
p = prevalence taken from a previous study, 34.1%^2^
q = i-p
e = margin of error, 6%

The calculated minimum sample size was 267. We have 183 total medical students in the college. The sample size for the finite population is calculated as:


$$\begin{array}{ll} \text{n} & =\frac{\text{n}}{1+\frac{\text{(n}-1)}{\text{N}}}\\ & =\frac{\text{267}}{1+\frac{\text{267}-1}{\text{183}}}\\ & =109 \end{array}$$

Where,

n'= adjusted sample size
N = finite population
n = calculated sample size

The calculated sample size was 109. However, 120 medical students were included in the study.

For a collection of lip print samples, the lips were first cleaned with wet tissue paper, and then a layer of red-coloured lipstick was applied. The red lipstick was used over the surface of the lips unidirectionally.

For proper sample collection, the participants were requested to stretch to make it even on the whole surface. Then cellophane tapes of appropriate length size are taken and stuck to the lipstick-applied lip, and removed carefully such that the desired grooves are well differentiated in the cello tape. After collection of the procedure, the lip of the participant is cleaned properly with the help of commercial wet tissue. The sample was collected and preserved properly. All of the samples were scanned with the help of a Cam-scanner (software) that would reproduce the original copy and compile it in a document. Then it was thoroughly investigated with the help of the software on the laptop. The lip prints were identified, and separated following the classification given by Suzuki and Tsuchihashi.^6^

Data were entered in Microsoft Excel 2007 and analyzed using IBM SPSS Statistics version 21.0. The point estimate was calculated at a 95% CI.

## RESULTS

**Figure 1:**
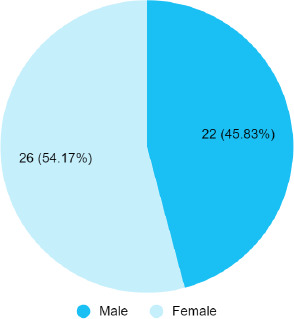
Gender-wise distribution of Type I lip patterns among medical students (n= 48).

Out of 120 medical students, the prevalence of Type I lip patterns was 48 (40%) (31.23-48.77, CI). Females were 26 (54.17%) with a female-to-male ratio of 1.18 (Figure 1).

## DISCUSSION

Among 120 students, the prevalence of Type I lip patterns among the medical students was 40%. It was higher in comparison with the study conducted in the same setting where prevalence of of Type I lip patterns was 34.1%.^2^ Similarly, other studies done in India have also reported it to be the most common type with a prevalence of 24% to 30%.^7,8^ However, other studies done in various populations such as Indian, Indo-Dravidian and Japanese found other types to be more prevalent.^9-11^

In this present study, the Type I lip print pattern was most common in both males and females similar to other studies done in Nepal.^1,2^ A study was done over cheiloscopy on gender discrimination in the Indian population also showed Type I as the predominant type in males while Type IV and Type V were predominant among all the regions of lips in females.^12^ Numerous researches have demonstrated that the lip print patterns formed reflected a population-wise dominance, or the majority of a specific lip print type in a given community.^13^

This study was conducted in a single center among small samples so the study might not be generalizable to a larger population. The influence of different factors was not evaluated as this was a cross-sectional study. Further research on lip print should use a material that does not undergo smudging or spoiling of the lip prints causing unidentifiable prints to avoid unnecessary workload.

## CONCLUSIONS

The prevalence of Type I lip pattern among medical students was higher compared to other studies done in similar settings. The type of lip print can be used

as a tool for identification. Since the grooves may not be finely appreciated in most cases in such studies, the investigator should extract and interpret lip prints properly.
